# Pupillary Responses Obey Emmert’s Law and Co-vary with Autistic Traits

**DOI:** 10.1007/s10803-020-04718-7

**Published:** 2020-10-21

**Authors:** Chiara Tortelli, Marco Turi, David C. Burr, Paola Binda

**Affiliations:** 1 Department of Surgical, Medical, Molecular and Critical Area Pathology, University of Pisa, Pisa, Italy; 2 Fondazione Stella Maris Mediterraneo, Chiaromonte, PZ Italy; 3 Department of Neuroscience, Psychology, Pharmacology and Child Health, University of Firenze, Florence, Italy; 4 Department of Translational Research on New Technologies in Medicine and Surgery, University of Pisa, Via San Zeno 31, 56123 Pisa, PI Italy

**Keywords:** Autistic traits, Pupillometry, Perceptual illusion, Individual differences

## Abstract

We measured the pupil response to a light stimulus subject to a size illusion and found that stimuli perceived as larger evoke a stronger pupillary response. The size illusion depends on combining retinal signals with contextual 3D information; contextual processing is thought to vary across individuals, being weaker in individuals with stronger autistic traits. Consistent with this theory, autistic traits correlated negatively with the magnitude of pupil modulations in our sample of neurotypical adults; however, psychophysical measurements of the illusion did not correlate with autistic traits, or with the pupil modulations. This shows that pupillometry provides an accurate objective index of complex perceptual processes, particularly useful for quantifying interindividual differences, and potentially more informative than standard psychophysical measures.

## Introduction

Although atypical perception is not a diagnostic criterion for Autism Spectrum Disorders (ASD), growing evidence shows that autistic individuals have different perceptual styles than neurotypicals. Perhaps the best known aspect of this consists of a preference for local details in children and adults with ASD, who often outperform controls in tasks requiring the discrimination of fine-grained visual features abstracted from their global context, like the embedded figure task or visual search tasks (Chouinard et al. 2016; Jolliffe and Baron-Cohen 1997; Shah and Frith 1983). Several theories have been developed to account for perceptual idiosyncrasies in ASD. These include the “Weak Central Coherence Theory” (Happe and Frith 2006), where the preference for focusing on details brings about a failure to extract (or, in more recent formulations, a preference to disregard) global “gestalt” cues (Chouinard et al. 2016; Happe and Frith 2006). The “Enhanced Perceptual Functioning theory”, posits that local preference results from overtraining of sensory function, which interferes with higher order operations necessary to capture the “gestalt” of sensory experience (Mottron et al. 2006). A more recent account links atypical autistic perception with Bayesian models of sensory integration. The key concept is that perception is a form of implicit inference, where sensory information is used to test hypotheses on the status of the world around us—hypotheses that we implicitly and automatically make based on a priori knowledge (Gregory 1980; Helmholtz and Southall 1962). Pellicano and Burr (2012) proposed that perception in ASD is less influenced by prior experience—it is more “data-driven”. A similar concept is at the basis of several other proposals that have been recently put forward (Friston et al. 2013; Lawson et al. 2014; Rosenberg et al. 2015; Sinha et al. 2014; van Boxtel and Lu 2013; Van de Cruys et al. 2014).

All these models would predict that autistic perception should be less susceptible to illusions. Illusions typically occur when one element is integrated into its global context, which can be informative and efficient in most real-world circumstances, but can be misleading in the peculiar settings that prompt illusions. If global information is underweighted in autism, it follows that an element may be seen “as is” irrespectively of its context, hence less illusorily (e.g. Happe 1996). Also, illusions can often result from a priori assumptions; if prior knowledge is underweighted in autism (as suggested by Pellicano and Burr 2012), it follows that perception may be generally less efficient, but paradoxically more veridical in limit cases that generate illusions.

In the face of this theoretical consensus, experimental approaches testing susceptibility to illusions in ASD have produced mixed results. Several studies compared behaviour in individuals with ASD and controls, and found evidence for reduced susceptibility to illusions in individuals with ASD (Bolte et al. 2007; Happe 1996; Mitchell et al. 2010). However, other studies have failed to detect significant differences in the strength of illusory effects between ASD and controls (Hoy et al. 2004; Manning et al. 2017; Milne and Scope 2008; Ropar and Mitchell 1999, 2001).

A variety of factors could account for the divergent findings. First, there are factors related to the selection criteria of participants, a common concern in clinical studies, particularly relevant for the highly heterogeneous class of ASDs. For example, different studies may have considered clinical samples with varying degrees of severity (Baron-Cohen and Wheelwright 2004; Belmonte et al. 2004; Happe and Frith 2006; Ring et al. 2008); comorbidities may have acted as confounding factors (Gillberg and Billstedt 2000), and the decision to match cases and controls based on chronological or mental age might also have mattered (Gori et al. 2016; Walter et al. 2009). Second, there are factors related to the psychophysical task. Any procedure for measuring illusions is sensitive to the instructions given to the participants and to the details of the procedure, which may well have differed in subtle ways across studies (Gori et al. 2016; Happe and Frith 2006). Moreover, compliance with the task is likely to be reduced in the ASD groups, given the high prevalence of cognitive disability, anxiety disorders and perseverative behaviours (Chouinard et al. 2013, 2016).

To mitigate these concerns, two strategies have been recently proposed. The first addresses the difficulty of studying clinical populations, and proposes to shift attention towards typically developed individuals that share features, or traits, with the clinical cases. There is a vast literature supporting a dimensional concept of autistic traits, distributed along a continuum across the whole population, of which the clinical sample forms an extreme (Bailey et al. 1995; Baron-Cohen et al. 2001; Chouinard et al. 2013, 2016; Constantino and Todd 2003; Piven 2001; Ruzich et al. 2015; Skuse et al. 2009; Wheelwright et al. 2010). A validated tool for quantification of these autistic traits is the Autistic Quotient Questionnaire, available in most languages for both adults (Baron-Cohen et al. 2001) and children (Auyeung et al. 2008). Using this tool, recent studies have reported a link between susceptibility to visual illusion and autistic traits in typical adults (Chouinard et al. 2013, 2016; Walter et al. 2009), suggesting that some but not all types of visual illusions (in which are involved different kind of perceptual integration) could be affected by the level of autistic traits.

The second strategy addresses the difficulties inherent in psychophysical techniques, aiming to develop objective measures to support the necessarily subjective psychophysical measures. Our group and others have proposed pupillometry. The diameter of the eye pupil is mainly affected by light and sympathetic tone, but also shows more subtle variations that reflect attentional and perceptual events. For example, a white disk elicits a stronger pupillary constriction when it is interpreted as a picture of the sun vs. the moon (Binda et al. 2013b; Naber and Nakayama 2013). Brightness illusions are also accompanied by enhanced constriction (Laeng and Endestad 2012; Zavagno et al. 2017). Even simply shifting covert attention to locations or surfaces/features with higher luminance is sufficient to induce a relative constriction (Binda and Murray 2015b; Binda et al. 2013a, 2014; Mathot et al. 2016, 2013; Turi et al. 2018), suggesting that tiny pupil size changes can track the focus of attention and the content of perception (Binda and Gamlin 2017; Binda and Murray 2015a; Mathot and Van der Stigchel 2015).

Recently both strategies has been combined, using pupillometry to index the preference for local elements vs. global configuration in association with autistic traits measured as AQ scores (Turi et al. 2018). This provided clear evidence that pupillometry reliably tracks inter-individual differences in perceptual styles: quickly and objectively, without interfering with spontaneous perceptual strategies.

In the present study we apply a similar logic, using pupillometry to index illusion-susceptibility, and relate it to autistic traits. Specifically, we applied a version of the Ponzo size illusion (Ponzo 1910) where the apparent size of an object changes illusorily with its apparent depth 3D. This illusion is a clear example of Emmert’s law (Boring 1940), according to which images of the same retinal size will look larger or smaller depending on their apparent distance, with nearer images appearing smaller and more distant images appearing larger, consistent with the conditions that would have cast that image. As the object (a small figurine) was brighter than the background, it is expected to evoke a pupillary constriction that scales with the actual size of the object. Combining pupillometry with psychophysics to examine a group of neurotypical adults, we asked: (1) whether pupil-constriction strength also scales with apparent object size when this is varied independently of actual size by 3D context; (2) whether the objective measure of illusion strength provided by pupillometry is tightly correlated with subjective measures obtained by psychophysical testing; and (3) whether between-participant variance of pupillometry and/or psychophysical estimates is associated with variance in autistic traits, estimated through the AQ scores.

Based on previous studies showing that pupillary responses are modulated by contextual information, we hypothesize that the pupillary response evoked by a light stimulus should be modulated by its perceived size, which in turn depends on its 3D context (Ponzo Illusion). We expect that the contextual effect should vary across individuals, being reduced in individuals with stronger autistic traits (higher AQ scores).

## Methods

### Compliance with Ethical Standards

None of the authors have conflicts of interest to declare. The research reported here involved human participants, who gave their written informed consent to the participation in this study. Experimental procedures were approved by the regional ethics committee [*Comitato Etico Pediatrico Regionale—Azienda Ospedaliero-Universitaria Meyer—Firenze (FI)* “under the protocol “Fusione di Informazioni Multisensoriali" v4/04.10.2018”] and were in accordance with the Declaration of Helsinki.

### Participants

50 neurotypical adults (33 females; mean age and standard error: 25.7 ± 4.0) were recruited for the study. All participants are university students and reported normal or corrected-to-normal vision, and no known neurological or medical condition.

### The Autism-Spectrum Quotient Questionnaire (AQ)

The AQ is a 50-item self-report questionnaire measuring tendency towards autistic traits (Baron-Cohen et al. 2001). Participants filled out an Italian version of the test (Ruta et al. 2012) on an on-line format at the end of the experimental session, before leaving the lab. Responses are made on a 4-point scale: ‘‘strongly agree’’, ‘‘slightly agree’’, ‘‘slightly disagree’’, and ‘‘strongly disagree’’ (in Italian). Items were scored as described in the original paper: 1 when the participant’s response was characteristic of ASD (slightly or strongly), 0 otherwise. The score can vary between 0 and 50, with higher scores indicating greater inclination towards autistic traits. All participants of our sample scored under 32, which is the cut-off over which a clinical assessment is recommended (Baron-Cohen et al. 2001). In our sample, the AQ scores ranged between 2 and 31 with a median score of 16.

### Apparatus and Stimuli

**Fig. 1:**
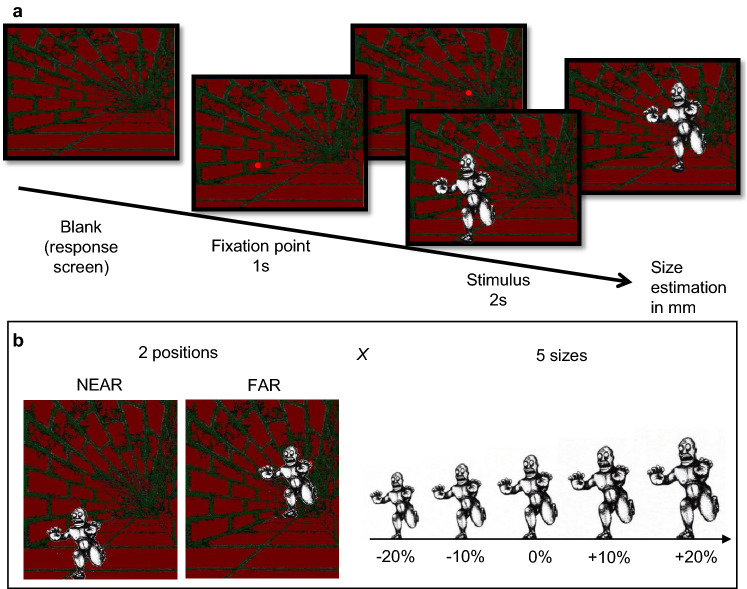
Stimuli and procedure. **a** Ponzo Illusion Task: timeline of the stimuli presentations. The stimulus was a white figurine displayed for 4 s within an illusory 3D corridor. Participants verbally estimated the size of the stimulus in millimeters. **b** The stimulus was presented in two experimental conditions, near and far positions of the illusory corridor, with 5 different psychical sizes (size varied of ± 20%, ± 10%, 0%; compared to the height of the original figurine, 7.8° of visual angle)

The experiment was performed in a quiet dark room (no lighting; windows obscured with shutters). Participants sat in an experimental boot inside thick black curtains that further shut off any ambient light. Thus, the only illumination was provided by the stimulus display, identical for all participants. This was a CRT monitor screen (40 × 30°, Barco Calibrator, resolution of 1024 × 768 pixels and a refresh rate of 120 Hz), placed at a distance of 57 cm from the participant’s head, which was stabilized by chin rest. Viewing was binocular. Stimuli were created by modifying a well-known example of the Ponzo Illusion. White figurines (with a luminance of 55 cd/m^2^) representing a monster were shown against a steady background creating a 3D context (corridor) with equiluminant red/green elements (green: 1.7 cd/m^2^; red: 2.0 cd/m^2^). Stimuli were generated with the PsychoPhysics Toolbox routines (Brainard 1997; Pelli 1997) for MATLAB (MATLAB r2010a, The Math Works) housed in a Mac Pro 4.1. Five figurines were used, with height varying between 6.2 and 9.3° in steps of 0.8° (the width varied proportionally). On each trial, a single figurine was presented, positioned to appear standing at the near-end or far-end of the corridor (see Fig. 1).

Two-dimensional eye position and pupil diameter were monitored at 500 Hz with an EyeLink 1000 system (SR Research) with infrared camera mounted below the screen, recording from the left eye. Pupil measures were calibrated by an artificial 4-mm pupil. Eye position recordings were linearized by a standard 9-point calibration routine performed at the beginning of each session (two sessions per participant). Synchronization between eye recordings and visual presentations was ensured by the Eyelink toolbox for MATLAB (Cornelissen et al. 2002).

### Procedure

Participants started the experiment with a brief training session on the size estimation task. They were shown the five figurines 20 times, always presented against a uniform black screen, and asked to voice their size estimate in millimeters. All participants understood the task and proceeded to the experimental session. This comprised 100 trials (5 figurine sizes, 2 figurine positions, and 10 repetitions of each combination), administered in two blocks of 50 trials. During a block, the background-corridor remained constantly visible. At the beginning of each trial, a fixation point (0.25° diameter) was presented at either the front or far-end of the corridor for 1 s, allowing participants to move their gaze to the location of the upcoming figurine. After this interval, the fixation point was extinguished and the figurine appeared at the same location, remaining on-screen for 4 s. During this time, fixation was not enforced (no fixation-point was visible); participants were encouraged to focus their attention on the entire figurine in order to estimate its size as precisely as possible, and to voice their estimate on extinction of the figurine. The experimenter entered the response by keyboard, automatically starting the following trial. Participants were asked to minimize blinking during the trial, postponing it to the inter-trial interval during which they voiced their responses.

### Analysis of Pupillometry and Eye-Tracking Data

Eye-tracking data were preprocessed using custom Matlab scripts that implemented the following steps:

1. Exclusion of the first trial in each block (due to the sudden appearance of the corridor against a completely dark background, which induced additional pupil constriction that contaminated the pupil light response to the figurine).
2. Identification and removal of gross artifacts: removal of time-points with unrealistically small or large pupil size (more than 1 mm from the median of the trial or < 0.1 mm, corresponding to blinks or other signal losses).
3. Identification and removal of finer artifacts: identification of samples where pupil size varied at unrealistically high speeds (> 2.5 mm per second, beyond the physiological range) and removal of the 20 ms epoch surrounding this disturbance.
4. Down-sampling of data at 10 Hz, by averaging the retained time-points in non-overlapping 100 ms windows. If no retained sample was present in a window, that window was set to “NaN” (MATLAB code for “not a number”).

Horizontal and vertical gaze position traces were transformed into deviations from screen center to degrees. Pupil traces were transformed into changes from baseline by subtracting the average pupil diameter in the first 200 ms after stimulus onset (i.e. during the latency of the pupillary light response).

**Fig. 2:**
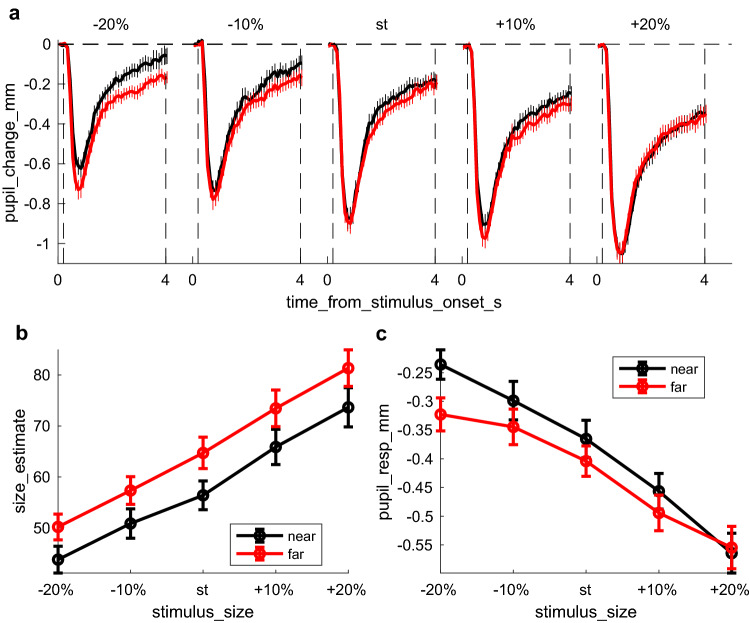
Ponzo illusion measured with perceptual reports and pupillary responses. **a** Timecourses of pupil change relative to baseline (first 200 ms from stimulus onset), plotted separately for the near (black) and far (red) conditions and for the five stimulus sizes (from ± 20% the standard size); dashed lines indicate the window over which pupil changes are averaged to compute the pupil response (whole trial duration except the initial 200 ms used to define pupil baseline). Thick lines show averages across participants, thin lines ± 1 s.e.m. **b** Mean magnitude estimates of the figurine size. Separate lines are for the near (black) and far (red) conditions and estimates are plotted against stimulus size, with error bars giving standard errors across participants. **c** Mean pupil responses (pupil change averaged in the window defined by dashed vertical lines in **a**, corresponding to the whole stimulus duration except the initial 200 ms used to define pupil baseline). Same format as in **b**

For statistical comparisons, we summarized the pupil change and gaze position traces by averaging over the stimulus presentation window (excluding the initial 200 ms used for baseline estimation). Due to the preprocessing described above, trials with blinks or artifacts included traces with several missing values; we excluded these from our analyses by eliminating all traces for which 40% or more of the 10 Hz samples were missing (mean ± s.e.m across participants: 20.4 ± 3.6% for a total of 921 trials across all participants). We verified that varying the values of any of these preprocessing parameters does not critically change the results (specifically, we verified that setting the maximum pupil deviation to 2 mm does not introduce the saturation effect seen in Fig. 2).

### Statistical Analysis

We used a linear-mixed-model approach to analyze data from individual trials. We modelled fixed effects for the figurine location (nominal variable with two levels: near/far) and figurine size (nominal variable with five levels), and a random effect to allow the intercept of the linear model to vary on a participant-by-participant basis. In addition, we separately modelled the interaction between figurine location (near/far) and AQ scores (real values, with as many levels as the scores we observed in our sample), again letting the intercept of the model vary across participants. Thus defined, the model allows for different participants having idiosyncratic response sizes, e.g. overall pupillary response amplitude, while fixing the relationship between AQ and the response difference across near/far figurine locations, which the model quantifies as the “AQ” × “near/far” interaction. The same models with the same trial selection (determined by the validity of pupil measurements) were applied to behavioral performance (size estimates) and gaze-behavior (pupil diameter and gaze deviations).

We complemented this analysis with a repeated-measures approach (mainly, for visualization purposes), computing average per-participant responses, analyzing these for figurine location and size, and correlating the results with the participants’ AQ scores through Pearson’s correlation coefficient. Significance of these statistics was evaluated using both p-values and log-transformed Bayes Factors (Wetzels and Wagenmakers 2012). The Bayes Factor is the ratio of the likelihood of the two models H1/H0, where H1 assumes a correlation between the two variables and H0 assumes no correlation. By convention, when the base 10 logarithm of the Bayes Factor (lgBF) > 0.5 is considered substantial evidence in favor of H1, and lgBF <  − 0.5 substantial evidence in favor of H0.

### Data Availability Statement

The data reported in this manuscript may be found at the following link: https://doi.org/10.5281/zenodo.3940543.

## Results

We tracked the pupillary response evoked by the presentation of a light stimulus (monster-like figurine) within an illusory 3D context: a corridor extending in depth. As expected from Emmert’s law, perceived size of the figurine depended on its apparent 3D location, with a relative size overestimation for the figurines at the far-end of the corridor (Fig. 2b). Importantly, pupillary light responses also varied with apparent 3D location (Fig. 2a, c): stronger pupillary responses were evoked by figurines at the far-end, which were matched in physical size but perceived as larger than figurines at the near-end.

We used a linear-mixed-model to evaluate the two effects statistically; we modelled the actual figurine size (five sizes) and its apparent 3D location (near/far) as fixed effects and added a random intercept to account for inter-individual variability of average responses (e.g. larger size estimate or pupil responses across all conditions). As expected both physical size of the figurines and their apparent 3D location independently affected behavioral size estimates (significant main effect of size: F(4,3921) = 592.26, p < 0.00001; significant difference between figurines at the far/near end of the corridor: F(1,3921) = 115.37, p < 0.00001; no significant interaction between figurine size and location: F(4,3921) = 1.55, p = 0.18). Pupillary responses were also significantly affected by 3D location, but the effect depended on figurine size (significant interaction between figurine size and location: F(4,3921) = 2.80, p = 0.024), possibly due to a saturation effect.

**Fig. 3:**
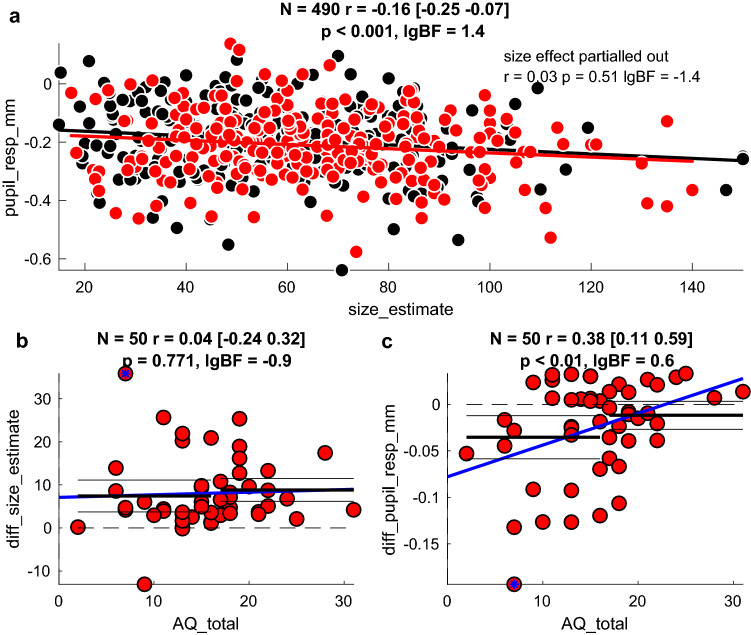
Association between illusion magnitude and Autistic Quotient scores. Correlation analyses: legends report the number of points entered the correlation analysis, the Pearson’s rho coefficient and its confidence interval; the associated p-value and lgBF, or the logarithm with base 10 of the Bayes Factor (see “Methods”). **a** Correlation between pupil responses and size estimates. Each participant is represented by a maximum of 10 points, one for each stimulus size and condition (missing points are for invalid trials/failed pupil measurements). Thick lines show the best fit linear regression across the data points of the corresponding color. After partialling out the effect of stimulus size, the correlation between pupil responses and size estimates becomes non-significant (text inset). **b** Psychophysical estimate of illusory size (pooled across stimulus size) plotted against AQ scores. Each participant is represented by 1 point, given by the subtraction of the mean response to figurines at the far-end minus the near-end of the corridor. The two measures were not correlated (see legend). The thick blue line shows the best fit linear regression across the data points and horizontal black horizontal lines (thick and thin) show the illusion size (mean and 95% confidence intervals) in participants with lower and higher AQ scores, defined by a median split. **c.** Pupillary response to illusory size (pooled across stimulus size) plotted against AQ scores. The correlation remains significant at p < 0.05 after removing the participant with the most extreme value (marked by the blue asterisk). The thick blue line shows the best fit linear regression and the black horizontal lines show the median split analyses: the pupil size modulation is non-significantly different from 0 for individuals with higher AQ scores (> 16, the median of our sample); for individuals with lower AQ scores, it is significantly below 0 (implying enhanced pupil constriction in response to figurines on the far-end of the corridor)

Thus, behavioral and pupillary responses were similarly affected by the 3D context, with figurines perceived as larger evoking greater pupil constrictions. Figure 3a plots the average response to each individual figurine (each of the 5 different physical sizes) at each apparent location (near and far), correlating pupillary responses with psychophysical reports. The actual variation of physical size is expected to introduce a negative correlation (because larger figurines will induce stronger pupillary constriction). Indeed, the observed correlation is weakly negative. Importantly, this correlation was entirely abolished after the effect of physical size of figurine was partialled out. This implies that illusory effects measured by perceptual reports and pupillary responses are independent.

Given that the two measures are statistically independent, we tested whether either correlated with autistic traits (AQ). Specifically, we tested the hypothesis that individuals with higher autistic traits would be less susceptible to context, so responses to figurines should be less affected by apparent 3D location. The distribution of AQ scores was consistent with those expected from a sample of neurotypical individuals (range 2–31; median: 16). We quantified the effect of 3D context as the difference between the average response to figurines at the far vs. near end of the corridor (pooling across figurine sizes). For behavioral responses, this value was almost always positive (as all but one individual was susceptible to the illusion), and was uncorrelated with AQ scores, (r = 0.04, lgBF =  − 0.94). This provides robust evidence supporting the lack of correlation between the behavioral size illusion effect and autistic traits (Fig. 3b). No further trends emerged when analyzing each figurine size individually, rather than pooling across sizes.

However, pupillary responses did correlate significantly with AQ scores (r = 0.38, lgBF = 0.62), implying that pupil responses are modulated by the 3D context (stronger for figurines at the far-end of the corridor) more in participants with low than high AQ scores (Fig. 3c). Given this correlation, we divided the participant sample into two groups with high and low AQ (compared with the median of 16), and analyzed each separately. Pupil modulations were significantly higher than zero only in the subsample with low AQ scores (95% confidence intervals do not encroach the y = 0 axis), but not for those with high AQ scores (95% confidence intervals embrace the y = 0 axis). When analyzing each figurine size individually, we found the strongest correlation for the smallest stimulus size, which gives the strongest effect (Fig. 2c).

We complemented this correlational analysis with a linear-mixed model approach with individual trial data modelling two fixed effects, the apparent 3D location of the figurine (near/far) and AQ scores (see “Methods”). For behavioral responses, this analysis showed a main effect only of “near/far” location (F(1,3927) = 22, p < 0.00001—note that the degrees of freedom differ from the previous analysis due to the different number of levels in the modelled effects). There was no effect of AQ and no “AQ” × “near/far” interaction. Conversely, for pupillary responses we found a significant “AQ” × “near/far” interaction (F(1,3927) = 12, p = 0.00057), consistent with a larger pupil response difference in the far vs. near condition in individuals with weaker autistic traits.

The same analysis approach was applied to other eye-tracking measurements. Baseline pupil diameter measured at stimulus onset (mean and s.e.m. across participants: 4.26 ± 0.11 mm) was not affected by any of the experimental factors (all F < 2.3 and all p-values > 0.05), and did not correlate with AQ (r = 0.05 [− 0.23 0.33] p = 0.712, lgBF =  − 0.93). Gaze position, on the other hand, faithfully reflected the different figurine locations. Figurines at the far-end of the corridor were higher and more to the right, and so was gaze position (main effect of figurine location on horizontal gaze: F(1,3921) = 1320, p < 0.00001; on vertical gaze: F(1,3921) = 2658, p < 0.00001). In addition, because the figurine “feet” were always on the “ground” irrespectively of its size, their center-of-mass moved higher for larger figurines, and so did vertical gaze position (F(4,3921) = 74, p < 0.00001). It may be argued that these small gaze deviations (about 4° in any direction) could impact on the pupil size differences. This is unlikely, given that the impact of experimental factors on pupil and gaze are qualitatively different: figurine size and location interact to affect the pupil, whereas they independently affect gaze. In addition, we found no correlation between the effects of figurine location (difference far–near) on pupil responses and on horizontal gaze (r =  − 0.02 [− 0.30 0.26] p = 0.890, lgBF =  − 0.95) and on vertical gaze (r = 0.09 [− 0.20 0.36] p = 0.545, lgBF =  − 0.88): both correlation coefficients are associated with a lgBF less than − 0.5, implying robust evidence against an association between gaze and pupil modulations. Finally, we examined the relationship between gaze position and AQ and found no reliable associations (far/near difference of horizontal gaze r = 0.05 [− 0.23 0.32] p = 0.731, lgBF =  − 0.93; vertical gaze: r = 0.06 [− 0.22 0.34] p = 0.658, lgBF =  − 0.91), confirming that pupil size modulation is selectively correlated with autistic traits. Finally, we explored the effect of gender across participants. We compared the effect of 3D context in females (the majority of our sample, 33) and males (N = 17) and found no significant gender effect in either behavioural size estimation [t(48) = 0.18; p = 0.86; lgBF = − 0.52] or pupil response [t(48) = 0.75; p = 0.46; lgBF = − 0.43].

## Discussion

Emmert’s law states that objects with the same retinal image will look larger if they appear to be located further away (Boring 1940). A clear example of this is the Ponzo Illusion (Ponzo 1910). Our first objective was to show that Emmert’s law is reflected in the magnitude of pupillary light responses, evoked by objects that change perceived size with perceived distance. We showed that bright stimuli cause significantly stronger pupillary constriction when they appear to be at the far end than at the near end of an illusory corridor. The effect of the illusion is not merely to change pupil size, but to increase or reduce the pupillary response to the light-stimuli in the direction expected from a change of their physical size, increased or reduced. This modulation cannot be explained by factors that are well known to affect pupil size, such as changes in focus and arousal. Optical focus was constant throughout the experiment, implying no expected “near response”—the pupillary constriction usually coupled with accommodation and convergence (Bharadwaj et al. 2011; Marg and Morgan 1949; Zhang et al. 1996). A residual illusory near response could in principle be generated by the apparent 3D context, predicting a steady pupil constriction for trials where the stimulus, hence fixation and focus, were on the near end of the corridor. However, we see no such modulation of baseline pupil size; what we find is a stimulus locked modulation of the pupillary light response (and a larger pupil constriction for trials where the stimulus was on the far end of the corridor). Finally, a variety of studies have demonstrated that arousal and cognitive/emotional load are accompanied by pupil dilation (Hess and Polt 1960; Kahneman and Beatty 1966), but none of these effects can explain pupil differences that emerge with identical settings and task requirements, simply changing the apparent 3D context of the stimulus. Our findings indicate that the pupillary light response is not a simple subcortical reflex; it is modulated by complex visual perceptual processes, which take 3D context and size constancy into account. This is in line with much recent evidence showing that pupil diameter depends on signals other than retinal illumination, tracking attentional focus and perceptual content (Binda and Gamlin 2017; Binda and Murray 2015a; Mathot and Van der Stigchel 2015). These effects suggest a ‘top-down’ modulation of the subcortical system controlling the pupillary light response. Such modulation likely involves multiple neural substrates, possibly including direct pre-frontal input to the pupillomotor circuit, as well as input from the visual cortex, in turn modulated by pre-frontal and parietal signals, projecting a cortical representation of visual stimuli to the pupillary circuit (Binda and Gamlin 2017; Ebitz and Moore 2017).

Neuroimaging work in humans (Chen et al. 2019; Fang et al. 2008; He et al. 2015; Murray et al. 2006; Schwarzkopf et al. 2011; Sperandio et al. 2012) and neurophysiological studies in non-human primates (Ni et al. 2014; Tanaka and Fujita 2015) has consistently shown that the Emmert’s law affects visual representations in the visual cortex, where the retinotopic projection of the stimulus is magnified or contracted depending on its apparent 3D location and therefore its illusory size changes. It is possible that the enlarged representation of the bright figurine in occipital cortex is fed into the circuit controlling pupil size, so that a larger stimulus area generates a stronger pupil-constrictor signal. At the same time, the pupillomotor circuit holds a representation of the actual amount of light-flux generated by the stimulus. This suggests that the final pupil constriction response results from the integration of two sources, direct retinal projection and feed-back cortical input, which converge to define a one-dimensional output variable: pupil size. It is easy to imagine how this combination might be progressively dominated by retinal signals as these become stronger, due to a larger or brighter light stimulus. This might explain our finding of a weaker 3D context effect on pupillary responses to larger stimuli.

Having established the sensitivity of pupillary light responses to illusory size changes, we asked whether estimates of illusion strength obtained through pupillometry are consistent with estimates obtained through psychophysical magnitude estimation. The perceptual reports reliably tracked actual size and, like pupillometry, showed a systematic effect of 3D context—as in previous reports of the Ponzo Illusion. However, inter-individual variability in pupillometric and psychophysical estimates of illusion susceptibility were statistically dissociable: their correlation was non-significant with inferential statistics, and it was significantly absent with Bayesian statistics.

Given the independence between pupillometry and psychophysics, we asked whether inter-individual variability in illusion susceptibility, estimated by either measure, was associated with variability in autistic traits across our sample of neurotypical participants. We found that psychophysical estimates of illusion strength were uncorrelated with scores on the Autistic Quotient (AQ) questionnaire, consistent with Chouinard et al. (2016) who also found no relationship between AQ scores and susceptibility to size illusions. This is also generally consistent with the numerous studies finding no differences in illusion susceptibility in individuals with ASD compared with controls (Hoy et al. 2004; Manning et al. 2017; Milne and Scope 2008; Ropar and Mitchell 1999, 2001). However, for illusion susceptibility indirectly estimated through the modulation of pupillary responses, we found a significant association with AQ scores. Pupillary modulations with 3D context were stronger in individuals with lower AQ scores, and were nearly absent in individuals with higher AQ scores, consistent with the concept of reduced illusion susceptibility in autism (Happe 1996).

There is growing interest in using objective indices, such as pupillometry, to quantify the peculiarities of autistic perception. Several studies have attempted to use the dynamics of the simple pupillary light response (evoked by an isolated light flash) to dissociate individuals with and without ASD (Daluwatte et al. 2013; Fan et al. 2009; Lynch et al. 2018; Nyström et al. 2015); among the many parameters that can be used to define such dynamics, several have been found to differ, but with little consensus across studies (Lynch 2018). Other studies have used pupillometry to index cognitive or emotional load, reporting differences between ASD and controls (Anderson and Colombo 2009; Blaser et al. 2014; Nuske et al. 2014a, b; Wagner et al. 2016). Notably, none of these studies has focused on perceptual idiosyncrasies or modulations of the pupillary light response. A recent exception is a study by Laeng et al. (2018), which used a very similar approach to what we propose here, and measured the modulation of pupillary light responses to illusory glare. This study failed to find differences between adults with ASD and controls. However, we know that different types of illusions rely on disparate mechanisms, at different levels of visual processing; numerous studies suggest that autistic traits impact primarily higher-level mechanisms, whereas lower-level processes are similar between ASD and controls (Maule et al. 2018; Pellicano et al. 2007; Turi et al. 2015). Thus, it is possible that illusory glare arises at an earlier level than the Ponzo illusion and size constancy mechanisms (the former requiring only the integration of local surround within the retinal image; the latter implying the construction of a whole 3D representation); this would suggest that complex phenomena like the Ponzo illusion may prove more successful than illusory glare in revealing differences between ASD and controls. Our present data allow only for speculation, given that we did not measure autistic individuals, but rather studied autistic traits in neurotypical individuals.

Our findings are coherent with two other recent reports (Pome et al. 2020; Turi et al. 2018), where autistic traits were associated with pupillometric indices of perceptual processing, not with psychophysical estimates. Turi et al. showed that pupillary modulations driven by the shift of attention during the exposure to an illusory bistable stimulus is highly predictive of AQ scores in neurotypicals—while no behavioral measure of bistability or attention distribution achieved any predictive power, or correlated with pupillometry indices (Turi et al. 2018). Pomè et al. showed that the repetition of priming colour led to faster behavioural responses and to weaker pupil-dilation responses; however, the reaction times and pupil-dilations were un-correlated across participants and only pupil-dilation correlated with AQ scores (Pomè et al. 2020).

This follows other examples of lack of correlation between pupillary light responses and perceptual responses. For example, Benedetto and Binda (2016) showed that both light sensitivity and pupillary light responses are reduced during saccadic eye movements, but the suppression effects are not correlated across individuals, or trials. Binda et al. also showed that pictures of the sun are rated brighter than control images of equal luminance, and generate stronger pupillary responses, but pupillary response strengths are un-correlated with brightness ratings (Binda et al. 2013b). This systematic lack of association between pupillary and perceptual responses may be explained by assuming that separate visual representations with independent noise sources underly the two responses—in analogy with the separation of visual representations for “perception” and “action” originally introduced by (Goodale and Milner 1992). This hypothesis was originally inspired by clinical observations of rare patients with localized cortical lesions, who showed inaccurate size perception (impaired size-constancy) either for perception or for action (grasping). More recently, this was confirmed in neurotypical individuals, in peculiar conditions where size constancy could fail in perception but hold for grasping (Chen et al. 2018), clearly suggesting that size constancy is under the control of different mechanisms for perception and action (Sperandio and Chouinard 2015). Similarly, the dissociation that we find between the behavioural and pupillary response may reflect existence of independent pathways that process visual information and integrate it with 3D context for the purpose of perception or for the purpose of action—motor, or perhaps pupillomotor. At this stage, this hypothesis is entirely speculative; it makes the interesting prediction that autistic features may be more readily assessed by testing the pathway connected with action (pupillomotor or otherwise motor responses, such as grasping, reaching etc.), rather than perception. Future studies may be able to empirically address this possibility.

In conclusion, our findings show that pupil responses provide an accurate objective index of complex perceptual processes. They are more effective than perceptual estimates and particularly useful for quantifying interindividual differences that could be also extended to clinical population in order to measure individual perceptual processes with an objective and non-invasive technique.
